# Special Issue “Novel Anti-Proliferative Agents”

**DOI:** 10.3390/ph16101437

**Published:** 2023-10-10

**Authors:** Valentina Onnis

**Affiliations:** Department of Life and Environmental Sciences, University of Cagliari, 09042 Monserrato, CA, Italy; vonnis@unica.it

Cancer is a disease that can affect any organ and spread to other nearby or distant organs. Cancer is the second most frequent cause of morbidity and mortality in industrialized countries. The American Cancer Society estimates that 1,958,310 new cancer cases and 609,820 cancer deaths will occur in the United States in 2023 [1]. For the same year, 1,261,990 cancer deaths were predicted in EU-27 countries [2]. The availability of anticancer drugs based on the study of oncogenes and tumor suppressors which are involved in the emergence of human cancers has reduced the death rate and increased both the quality of life and life expectancy of tumor patients [3,4,5,6]. However, the failure of cancer therapies is still an urgent challenge due to reactions to existing treatments and multidrug chemoresistance [7,8,9].

In this Special Issue, different studies were reported relating to kinase inhibitors. Among antitumoral drugs, small molecule inhibitors of epidermal growth factor receptors (EGFR), including Erlotinib and its analogs [10], as well as monoclonal antibodies (e.g., cetuximab, necitumumab), are used in the therapy of non-small-cell lung cancer (NSCLC), breast, colon, pancreatic and thyroid cancer. Recently, studies have identified gene mutations targeting the kinase domain of the EGFR that are related to the response to inhibitors. Most EGFR mutations predict a higher benefit from treatment compared with wild-type receptors and are correlated with clinical features related to better outcomes; some EGFR mutations, however, confer drug resistance [11]. In this Special Issue, Youssif and co-workers described thiazole [12], purine/pteridine [13] and quinolone derivatives [14], dual EGFR/BRAFV600E inhibitors, as potential drugs in resistant NSCLC, in which BRAF mutation can cause resistance, even through EGFR stimulation. The same authors also described indole derivatives [15] as inducing apoptosis by EGFR and CDK2 dual inhibition. In NSCLC, both EGFR and its mutations, L858R/T790M, are overexpressed. New idantoine derivatives have been reported by Beshr and collaborators as potent inhibitors of these kinases [16].

In this Special Issue, two studies were dedicated to altering the tumor microenvironment [17,18] as a target for antiproliferative drugs. Death-associated protein kinase 1 (DAPK-1) is a positive mediator of gamma interferon-induced programmed cell death, and the loss- and gain-of-function of DAPK1 is associated with various cancer and neurodegenerative diseases, respectively [19]. Roh and collaborators reported on the anti-proliferative activity of aryl carboxamide derivatives acting as DAPK-1 inhibitors [20]. The c-Myc oncogene is a master regulator that has a very important role in regulating the transformed phenotype. The effects induced by c-Myc can occur either as a primary oncogene, which is activated by amplification or translocation, or as a downstream effect of other activated oncogenes. c-Myc is expressed in multiple types of cancer, comprising head and neck squamous cell carcinoma where it plays a fundamental role in tumor prognosis [21]. Diomede and collaborators underlined the functional relevance of c-Myc and HIF-Myc on oral squamous cell carcinoma (OSCC). In particular, their results indicated that c-Myc, c-Jun, Bcl-2, hypoxia inducible factor-1α (HIF-1α), vascular endothelial growth factor, matrix metalloproteinase-9, ERK 1/2 and pERK1/2 were overexpressed in OSCC [22]. In tumor cells, HIF-1α is activated by a deficient oxygen supply. HIF-1α activates the Carbonic Anhydrase (CA) IX and XII genes, leading to an improved resistance from tumoral cells to the acidic extracellular environment [23]. Shchekotikhin and collaborators described indoline-5-sulfonamide derivatives as CAIX and CAXII inhibitors exhibiting hypoxic selectivity, suppressing the growth of MCF7 cells and causing partial inhibition of hypoxia-induced CA IX expression in A431 skin cancer cells [24].

Another approach to contrast tumoral cell proliferation is acting in the mitotic stage of the cell cycle with microtubule binding agents [25] and telomerase inhibitors [26]. In this Special Issue, Viola and collaborators described 2-anilino-triazolopyrimidines as tubuluin polymerization inhibitors [27], Pérez-Pérez and collaborators reported salicylamides as affecting tubulin polymerization and/or STAT3 phosphorylation [28], Shakeel and collaborators studied pyrazole hybrid chalcones that arrested the cell cycle, induced apoptosis in a dose-dependent manner and inhibited the polymerization of tubulin [29], and El-Hamamsy and collaborators reported on selective non-nucleoside potent telomerase inhibitor BIBR1532 derivatives that were demonstrated to inhibit telomerase inside living cancer cells [30].

The methyltransferase-like proteins 3 (METTL3) and 14 (METTL14) in cancers have been shown to be closely associated with the proliferation, apoptosis, metastasis and differentiation processes in the progression of various human cancers [31,32]. In this Special Issue, Kim and collaborators reported on Eltrombopag. This compound exhibited selective inhibitory activity in the most active catalytic form of the METTL3–14 complex, interacting at a putative allosteric binding site in METTL3 [33].

The Wnt/β-catenin pathway has been identified as one of the most important oncogenic signaling pathways related to immune evasion [34,35]. In this Special Issue, Kadletz-Wanke and collaborators reported that the inhibitor of the CBP/Beta-Catenin interaction ICG-001 produces cytotoxic and anti-migratory effects on human papillomavirus-positive head and neck squamous cell carcinoma [36].

In this Special Issue, the antiproliferative or cytotoxic activity of various small synthetic, natural-derived molecules and their hybrids was discussed. Kim and collaborators reported on phenylisoquinoline derivatives endowed with antiproliferative activity against MDA-MB-231, HeLa and HepG2 cancer cell lines [37]. Ammazzalorso and collaborators described the antiproliferative activity of benzothiazole derivatives on AsPC-1, Capan-2 and BxPC-3 pancreatic cancer cell lines [38]. Tuyun and collaborators reported that plastoquinone derivatives exerted notable cytotoxicity toward colon cancer HCT-116 and breast MCF-7 cells compared to cisplatin [39]. Abdala-Díaz and collaborators reported that ulvan polysaccharides obtained from *Ulva rigida* demonstrated antiproliferative activity on the HCT-116 tumor cell line [40]. Lokeshwar and collaborators demonstrated that the conjugation of the poor bioavailable antiproliferative compounds Curcumin and dichloroacetate by aminoacidic linkers improves bioavailability and reduced the growth of several breast cancer cell lines and tumor growth and metastasis on transgenic mouse breast cancer (BC) and metastatic BC tumor-bearing mice without showing signs of toxicity [41].

Curcumine and other naturally occurring agents have been proposed as cancer chemopreventive agents and were proposed for treating human malignancy [42,43]. Natural products have played an important role in chemotherapy and chemoprevention by providing antitumor drugs such as camptothecin, doxorubicin, paclitaxel, vinblastine and vincristine, as well as understanding the cellular and molecular mechanisms underlying antitumor activity. Natural products are a rich source of bioactive molecules endowed by a great variety of chemical scaffolds. Natural compounds are often used in traditional medicine and used to build semisynthetic molecules with improved biological properties [44,45,46]. In this context, Shuvalov and collaborators reviewed the information about plants and mushrooms, as well as their active compounds with antitumor properties. Plants and mushrooms were divided based on the regions where they are used in ethnomedicine to treat malignancies [47].

In conclusion, this Special Issue presented recent findings on antiproliferative compounds and highlighted possible routes to discover new drugs against cancer. I hope that this Special Issue can be of inspiration to readers working in cancer research and stimulate the distinct fields involved in the continuous search for novel strategies for anticancer therapy. Finally, I would like to thank all of the authors and reviewers for their valuable contributions.
